# Paying Patients and Doctors' Fees

**Published:** 1921-02-12

**Authors:** 


### PAYING PATIENTS AND DOCTORS' FEES.

There has always been a distinction between the positions of the hospital patient who contributes towards the cost of his maintenance and of the patient who pays a rate above the average cost of the whole of the patients of the hospital. The first is more distinctly an object of charity than the other, whose position is in many respects that of one who is paying his way. An unpaid medical staff is clearly in the second instance giving something to the hospital for which payment is taken from the patient. This principle is recognised in the Great Northern Hospital scheme, which, with regard to the private wards with beds at four and a-half to six guineas a week, provides for separate and further payment of physicians and surgeons. There is nothing new about this, for many hospitals have for a long time received patients on these terms. (See p. 458.)

But when it comes to payment of the senior medical staff of the hospital in respect of patients who make contributions of any amount, whether below or above the cost of maintenance, the question is not so easily dealt with. Despite what has been said about physicians and surgeons no longer regarding the experience and reputation gained at the hospitals as adequate compensation for voluntary services, there are undoubtedly some among these gentlemen to whom such payment gives a feeling of repugnance which further complicates the matter. But this is a commercial and unsentimental age, and such a body as the British Medical Association, whose members are of course not chiefly men who hold hospital appointments, keeps well in the foreground the question of the payment of hospital staffs for patients who themselves pay, or who have as responsible for them certain Government Departments. The claim for payment no doubt is influenced by the element of competition arising between the institution and the general practitioner, and if only from this point of view can fairly be argued.

But in a sense the paid senior doctor becomes an employé just as much as the matron, secretary, or pharmacist, and he must lose the prestige that his entirely honorary position gave him with regard to an independent voice in the affairs of the hospital. Nor, should his status become too distinctly commercialised, can he hope to retain as an important and valued feature of his position practical immunity from dismissal and a clearly defined tenure of office. His position, if payment is forced upon him, is disagreeable as well as difficult.

In a number of institutions the problem has been solved in what we venture to think is a satisfactory and graceful manner. In many hospitals there are certain branches of the work which cannot, chiefly for want of funds, adequately be supported out of hospital revenue. As an instance, research may be mentioned. It is desirable to retain the services of a good research director or pathologist, and an extra hundred or two augmenting what the hospital can afford to pay will keep a good man and give distinct aid to the reputation of the hospital. A little imagination will readily add other instances of what can be done with moderate unofficial funds. We suggest that it would be fair to deduct a percentage of what the patients pay or of what is paid for them, and to place the resulting sum at the disposal of the honorary medical staff. We do not think the hospital will lose by such an arrangement; indeed, it is conceivable that it will gain materially in the long run.

